# Enhancement of prostaglandin D_2_-D prostanoid 1 signaling reduces intestinal permeability by stimulating mucus secretion

**DOI:** 10.3389/fimmu.2023.1276852

**Published:** 2023-10-24

**Authors:** Akane Hayashi, Naoaki Sakamoto, Koji Kobayashi, Takahisa Murata

**Affiliations:** ^1^ Animal Radiology, Graduate School of Agricultural and Life Sciences, The University of Tokyo, Tokyo, Japan; ^2^ Food and Animal Systemics, Graduate School of Agricultural and Life Sciences, The University of Tokyo, Tokyo, Japan; ^3^ Veterinary Pharmacology, Graduate School of Agricultural and Life Sciences, The University of Tokyo, Tokyo, Japan

**Keywords:** D prostanoid receptor, prostaglandin D_2_, intestinal barrier, intestinal permeability, mucin

## Abstract

**Introduction:**

The intestinal barrier plays a crucial role in distinguishing foods from toxins. Prostaglandin D_2_ (PGD_2_) is one of the lipid-derived autacoids synthesized from cell membrane-derived arachidonic acid. We previously reported that pharmacological stimulation of PGD_2_ receptor, D prostanoid 1 (DP1) attenuated the symptoms of azoxymethane/dextran sodium sulfate-induced colitis and ovalbumin-induced food allergy in mouse models. These observations suggested that DP1 stimulation protects the intestinal barrier. The present study aimed to uncover the effects of DP1 stimulation on intestinal barrier function and elucidate the underlying mechanisms.

**Materials and methods:**

Intestinal permeability was assessed in mice by measuring the transfer of orally administered fluorescein isothiocyanate-dextran (40 kDa) into the blood. The DP1 agonist BW245C (1 mg/kg) was administered 10 min prior to dextran administration. The intestinal permeability was confirmed using the *ex vivo* everted sac method. Tight junction integrity was evaluated *in vitro* by measuring the transepithelial electrical resistance (TER) in the human intestinal epithelial cell line Caco-2. Mucus secretion was assessed by observing Alcian Blue-stained intestinal sections.

**Results:**

Pharmacological DP1 stimulation reduced intestinal permeability both *in vivo* and *ex vivo*. Immunohistochemical staining showed that DP1 was strongly expressed on the apical side of the epithelial cells. DP1 stimulation did not affect TER *in vitro* but induced mucus secretion from goblet cells. Mucus removal by a mucolytic agent N-acetyl-l-cysteine canceled the inhibition of intestinal permeability by DP1 stimulation.

**Conclusion:**

These observations suggest that pharmacological DP1 stimulation decreases intestinal permeability by stimulating mucus secretion.

## 1 Introduction

The intestinal tract digests food and absorbs nutrients. The intestinal epithelium forms a barrier distinguishing food from toxins. This barrier is mainly composed of epithelial and mucus layers (1). In the epithelial layer, tight junctions seal the paracellular space between epithelial cells and limit paracellular transport, except for that of small molecules and solutes. The mucus layer covers the epithelial cells and prevents interactions between the luminal contents and tissue. When the barrier is disrupted, foreign substances, including food allergens and bacterial toxins, invade tissues and the bloodstream. This condition is called “increased intestinal permeability” or “leaky gut”, which promotes the pathogenesis of various diseases, including inflammatory bowel disease (IBD) and food allergies (2, 3). Therefore, many researches have focused on factors regulating the intestinal barrier to find promising therapeutic targets for these diseases (4).

Cytokines and autacoids increase or decrease paracellular permeability by regulating the expression and intracellular localization of tight junction proteins. Raju et al. revealed that the intraperitoneal administration of interleukin-13 increased intestinal permeability by upregulating the expression of the tight junction protein claudin-2 in mice (5). Pochard et al. showed that intraperitoneal administration of synthetic prostaglandin (PG) I_2_ decreases intestinal permeability by increasing the membrane localization of the tight junction protein occludin (6). In addition, several autacoids promote mucus secretion from goblet cells. Abigail et al. showed that administration of serotonin or PGE_2_ to dissected mouse intestines promoted mucus secretion from goblet cells (7). Despite the increasing number of studies on intestinal barrier function, few drugs are currently available for regulating intestinal permeability.

PGD_2_ is a lipid-derived autacoid synthesized from cell membrane-derived arachidonic acid. This lipid exerts its biological function through two G-protein-coupled receptors, D prostanoid 1 (DP1), and chemoattractant receptor-homologous molecule expressed on Th2 cells (CRTH2). We previously reported that pharmacological DP1 stimulation is beneficial for azoxymethane/dextran sodium sulfate-induced intestinal inflammation (8) and ovalbumin-induced allergic inflammation (9). There are two main exacerbating factors in intestinal inflammation and allergic reactions: immune cell infiltration and activation and intestinal barrier disruption (10, 11). In a food allergy model, enhancement of DP1 suppressed mast cell infiltration and degranulation (9). On the other hand, there is another possibility that PGD_2_ suppresses these pathologies by strengthening the intestinal barrier. The present study aimed to examine whether enhancement of DP1 reduces the intestinal permeability and reveal its underlying mechanism 40 kDa.

## 2 Materials and methods

### 2.1 Animals

Thirty-five C57BL/6J mice (14 males and 21 females, purchased from Charles River Laboratories Japan, Inc., Yokohama, Japan) were used at eight to twelve weeks of age. All experiments used age- and sex-matched controls. Mice were housed under 12-hour dark/light cycle and given ad libitum access to water and feed (MF, Oriental Yeast Co., Tokyo, Japan). All the experiments were approved by the Institutional Animal Care and Use Committee of the University of Tokyo (P18-069 and P21-036).

### 2.2 *In vivo* measurement of intestinal permeability

*In vivo* intestinal permeability was measured by modifying the previously described methods (12, 13). Briefly, mice were fasted for 4 hr and orally administered fluorescein isothiocyanate-dextran 40 kDa (FD40; TdB lab, 20 mg/200 µl saline). Blood samples were collected 0.5, 1, 2 and 4 hr after the FD40 administration. BW245C (1 mg/kg, Cayman Chemical, Ann Arbor, MI, USA) was intraperitoneally administered 10 min prior to the oral administration of FD40. The fluorescence signal of the serum was measured with excitation at 485 nm and emission at 530 nm using ARVO X2 Multimode Plate Reader (PerkinElmer, Waltham, MA, USA).

### 2.3 *In vivo* analysis of mucus secretion

Mice were fasted for overnight and intraperitoneally administered BW245C (1 mg/kg) 15 min before the tissue collection. Swiss roll was prepared as previously described (14, 15) and fixed in 4% paraformaldehyde. Intestinal segments without flushing were fixed in methanol-Carnoy’s solution (methanol: chloroform: acetic acid = 6: 3: 1). Samples were embedded in paraffin and cut into 4-µm sections. The deparaffinized sections were immersed into 3% acetic acid and stained with Alcian Blue solution (pH 2.5, FUJIFILM Wako, Osaka, Japan). Several sections were counter-stained with hematoxylin (FUJIFILM Wako). Sections were observed using BZ-X700 microscope (Keyence, Kyoto, Japan). The area of Alcian Blue-positive cells and the whole tissue were measured using imageJ. The ratio of the area of Alcian Blue positive cells to the area of whole intestinal tissues in a Swiss roll was represented.

### 2.4 *Ex vivo* measurement of intestinal permeability

*Ex vivo* intestinal permeability was measured by modifying the previously described methods (12, 16). Jejunum and ileum samples (5 cm length each) were everted inside out to make sacs. The intestinal sacs were injected with Krebs buffer (KHBB, jejunum: 300 µl, ileum: 200 µl), and placed in the glass tube with KHBB (2 ml, aerated with 95% O_2_ and 5% CO_2_). BW245C (1 µM, final) was administered to each tube, and after 10 min, FD40 (20 µg/ml, final) was administered. After 30 min incubation, fluids inside the samples were collected. The fluorescence signal of the sample was measured as described above. The permeability was calculated as “Absorbed FD40” from mucosa to serosa using the following formula:


$$\begin{array}{c} “\text{Absorbed FD}40(µ\text{g}/\text{c}{\text{m}}^{2}/\text{min})”=\;\left(\text{FD}40\text{ concentration inside the sac }\times \text{ injected volume}\right)/\\ \left(\text{mucosal surface area}*\text{ × }30\;\text{min}\right) \end{array}$$

*Mucosal surface area = π × length of the sac × diameter of the sac

In some experiments, intestinal sacs were made after incubation with BW245C (1 µM) for 10 min following incubation with 150 µl of luminal 10% N-acetyl-l-cysteine (NAC; Sigma-Aldrich, St. Louis, MO, USA) for 10 min. Some of these samples were used for histological analysis.

### 2.5 Immunohistochemical staining of DP1

Ileum samples were fixed in 4% paraformaldehyde and embedded in paraffin. The samples were cut into 4-µm sections. The deparaffinized sections were immersed into 10 mM sodium citrate solution buffer (pH 6.0) with 0.1% trypsin and 0.1% CaCl_2_ for 15 min. The samples were immersed into 30% H_2_O_2_ in MeOH and permeabilized/blocked in PBS with 0.05% Triton-X and 5% NGS for 30 min. The sections were incubated with rabbit anti-mouse DP1 antibody (1:200, Ann Arbor) for overnight at 4°C. The sections were then labelled with biotinated goat anti-rabbit antibody (1:500) for 2 hr at room temperature (RT). After incubation with Avidin-biotin complex solution (VectorLaboratories, Burlingame, CA, USA) for 30 min at RT, sections were colored by incubating with 50 mM Tris buffer with 3,3’-diaminobenzidine (200 mg/mL) and hydrogen peroxidase (0.03%). Samples were co-stained with Alcian Blue or hematoxylin.

### 2.6 *In vitro* measurement of transepithelial electric resistance

Caco-2 was provided by Riken BRC (Tsukuba, Japan, RCB0988). These cells were cultured in Minimum Essential Media (Gibco, Thermo Fisher Scientific, Waltham, MA, USA) supplemented with 20% Fetal Bovine Serum and 1% non-essential amino acid (Gibco, Thermo Fisher Scientific, Waltham, MA, USA). Five-thousand cells were seeded in a 20 mm^2^ well with electrodes and incubated for 28 hr. BW245C (1 µM) was administered to the cells and TER was measured using the xCELLigence Real-Time Cell Analyzer System (Roshe, Basel, Switzerland). The value of TER was represented as the ratio to the value before the BW245C administration.

### 2.7 RT-PCR of DP1

Cells were homogenized in Trizol reagent and total RNA was extracted according to the manufacturer’s instructions. Extracted RNA was reverse-transcribed into cDNA using ReverTra Ace (TOYOBO, Osaka, Japan). The transcribed cDNA was combined with KOD SYBR qPCR Mix (TOYOBO) and specific primers as follows: *18S* forward 5’- CGTTCTTAGTTGGTGGAGCG -3’ and reverse 5’- AACGCCACTTGTCCCTCTAA -3’ (product size: 127); *DP1* forward 5’- TTCTACCGACGGCACATCAC -3’ and reverse 5’- GTACTGCACGAACTTCCCGA -3’ (product size: 117). Conventional PCR was performed as the following protocol: 1 cycle of 95°C for 1 min followed by 45 cycles of 95°C for 15 sec and 59°C for 1 min.

### 2.8 Data processing

The data were shown as mean ± SEM. Two-group comparisons were analyzed by Student’s *t*-test using excel. Multiple-group comparisons of *in vivo* measurement of intestinal permeability was analyzed by two-way ANOVA followed by Tukey’s test using EZR (17). Statistically significance was determined when *p*-value is less than 0.05.

## 3 Results

**Figure 1:**
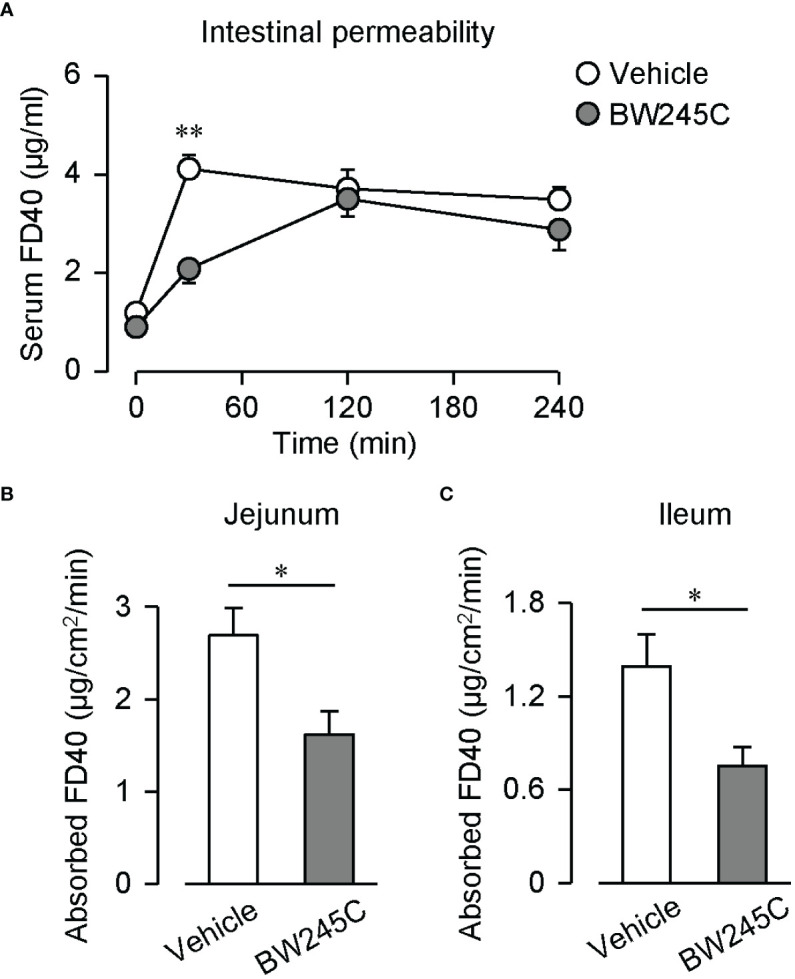
DP stimulation decreased intestinal permeability. **(A)** Serum FD40 level of vehicle and BW245C-treated mice after oral administration of FD40 (20 mg, n=5 each). Vehicle or BW245C (1 mg/kg) was administered 10 min before the FD40 administration. Data are represented as mean ± SEM. **(B, C)** Absorbed FD40 was measured *ex vivo* using everted intestinal sacs. Vehicle or BW245C (1 μM) was applied 10 min before the FD40 administration. The data of **(B)** jejunum and **(C)** ileum (n=6-8) were shown. Data are represented as mean ± SEM. *p<0.05, **p<0.01 compared with vehicle-treated group.

We assessed intestinal permeability by measuring the transfer of orally administered fluorescein isothiocyanate-dextran (FD40) into the blood. The administration of BW245C (1 mg/kg) decreased serum FD40 levels 30 min after administration (**Figure 1A**, vehicle: 4.11 ± 0.29 µg/mL, BW245C: 2.08 ± 0.29 µg/mL). These levels were comparable at 120 min and 240 min. Intestinal permeability was evaluated using an *ex vivo* method using an everted sac model. BW245C (1 μM) administration significantly decreased the absorbed FD40 in both jejunum and ileum (**Figures 1B, C**, jejunum; vehicle: 2.69 ± 0.30 µg/cm^2^/min, BW245C: 1.62 ± 0.25 µg/cm^2^/min, ileum; vehicle: 1.39 ± 0.21 µg/cm^2^/min, BW245C: 0.75 ± 0.12 µg/cm^2^/min).

**Figure 2:**
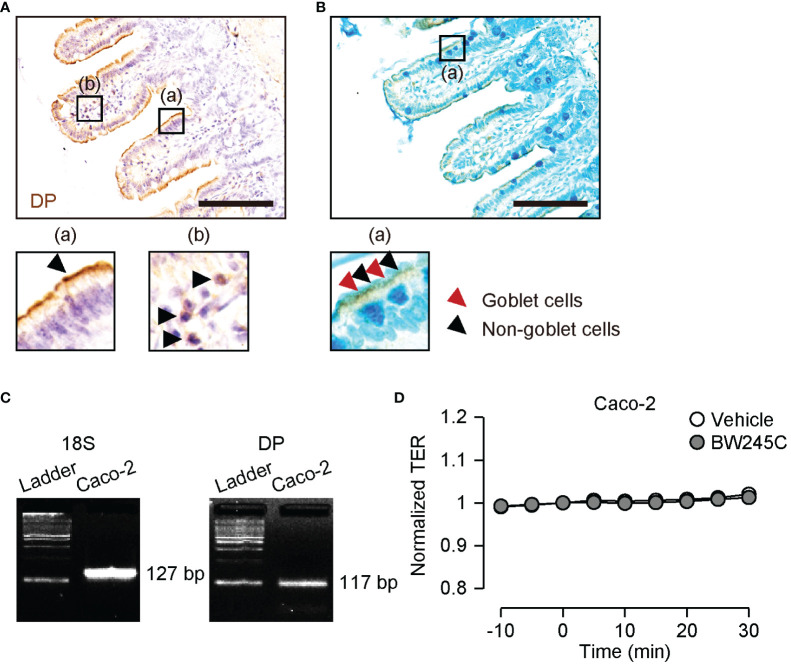
Expression and function of DP in intestinal epithelial cells. **(A, B)** Representative pictures of immunohistochemical staining of DP (brown) with hematoxylin **(A)** or with Alcian Blue **(B)** in ileum. The magnified pictures were shown in (a) and (b). **(A)** (a) epithelial site and (b) lamina propria. The arrowheads show epithelial cells (a) and immune cells (b). **(B)** (a) The arrowheads show goblet cells (red) and non-goblet epithelial cells (black). Scale bar = 100 µm. **(C)** Representative pictures of mRNA expression of 18S ribosomal RNA (18S, left panel) and DP (right panel) on Caco-2. **(D)** Change of TER of Caco-2 after vehicle or BW245C (1 μM) administration (n=4-8). Data are represented as mean ± SEM.

We previously showed that DP1 is strongly expressed in intestinal epithelial cells in a mouse model of food allergy (9). Immunohistochemical staining of DP1 showed that DP1 was strongly expressed on the apical side of epithelial cells in naïve mice [**Figure 2A** (a)]. Immune cells in the lamina propria also expressed DP1 [**Figure 2A** (b)]. Co-staining with Alcian Blue revealed that both goblet and non-goblet epithelial cells expressed DP1 [**Figure 2B** (a), red and black arrowheads, respectively].

Transepithelial electrical resistance (TER) is generally used to assess tight junction integrity. We measured the TER of the human intestinal epithelial cell line Caco-2. DP1 expression in Caco-2 cells was confirmed by RT-PCR (**Figure 2C**). Neither vehicle nor BW245C (1 μM) treatment affected TER during the time course (**Figure 2D**). This result indicates that BW245C decreases intestinal permeability through a mechanism other than the enhancement of tight junction integrity.

**Figure 3:**
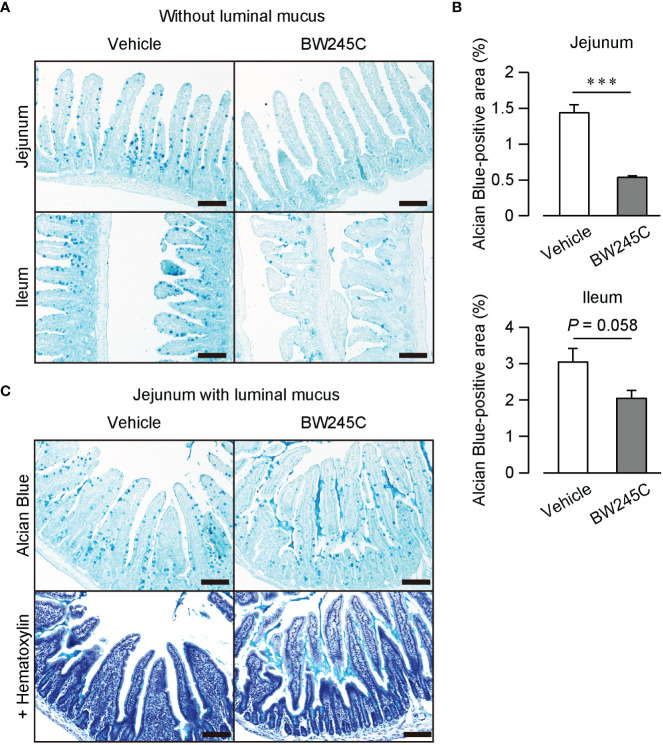
DP stimulation promoted mucus secretion *in vivo.* Representative pictures of Alcian Blue-stained sections of intestinal tissue of vehicle or BW245C treated mice. Vehicle or BW245C (1 mg/kg) was intraperitoneally administered 15 min before the sample collection. **(A)** Section without luminal mucus. Samples were fixed after flushing out intestinal contents Upper panel: jejunum, lower panel: ileum. **(B)** The ratio of Alcian Blue-positive area to the whole intestinal tissue area (n=4 each). **(C)** Section with luminal mucus. Samples were fixed without contents flushing out. Upper panel: with hematoxylin, lower panel: without hematoxylin. Scale bar = 100 µm. Data are represented as mean ± SEM. ***p<0.001 compared with vehicle-treated group.

Kuver et al. showed that an increase in the intracellular cAMP concentration leads to mucus secretion (18). We hypothesized that DP1, a Gs-coupled GPCR, induces mucus secretion. Alcian Blue staining of intestinal sections revealed numerous mucus-containing goblet cells in vehicle-treated mice (**Figure 3A**). The administration of BW245C (1 mg/kg) decreased the number of Alcian Blue-positive cells (**Figure 3A**). BW245C administration decreased the Alcian Blue-positive area in jejunum and tended to decrease in the ileum (**Figure 3B**, jejunum; vehicle: 1.44 ± 0.11%, BW245C: 0.54 ± 0.03%, ileum; vehicle: 3.05 ± 0.37%, BW245C: 2.05 ± 0.22%). A small amount of mucus was observed in the lumen of vehicle-treated mice (**Figure 3C**). BW245C administration increased the amount of luminal mucus, and this secreted mucus filled the space between the villi (**Figure 3C**). These results indicate that DP1 stimulation decreases intestinal permeability by inducing mucus secretion from goblet cells.

**Figure 4:**
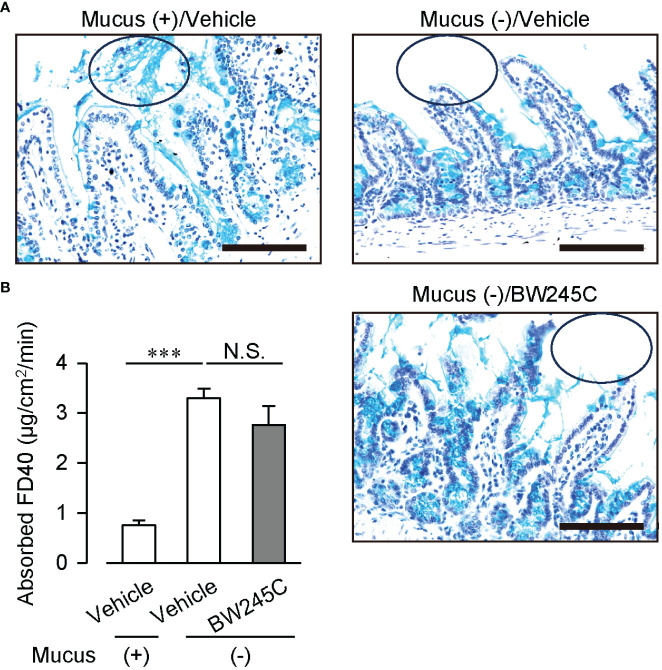
Mucus removement canceled DP-induced decrease of intestinal permeability *ex vivo.* Absorbed FD40 was measured using vehicle or BW245C (1 μM) -treated intestinal sacs with or without mucus removal by 10% NAC. Samples were incubated with vehicle or BW245C for 10 min followed by NAC treatment. **(A)** Representative pictures of Alcian Blue and hematoxylin staining of ileum used in the experiment. The circle shows luminal mucus. Scale bar = 100 µm. **(B)** Absorbed FD40 of ileum (n= 5-9) was shown. Data are represented as mean ± SEM. ***p<0.001 compared with mucus (+) vehicle-treated group. N.S., not significant.

We confirmed the effect of mucus secretion by BW245C on intestinal permeability. Treatment with a mucolytic agent N-acetyl cysteine (NAC) successfully removed the luminal mucus (**Figure 4A**). This removement of mucus increased the absorbed FD40 (**Figure 4B**, mucus (+); vehicle:0.75 ± 0.10 µg/cm^2^/min, mucus (–); vehicle: 3.30 ± 0.20 µg/cm^2^/min), and inhibited BW245C-induced decrease in absorbed FD40 (**Figure 4B**, mucus (-); BW245C: 2.76 ± 0.38 µg/cm^2^/min). These observations suggest that BW245C-induced mucus secretion is indispensable for decreasing intestinal permeability.

## 4 Discussion

In this study, we demonstrated that the administration of a DP1 agonist decreased intestinal permeability, potentially through stimulation of mucus secretion from goblet cells. The results of immunostaining and *ex vivo* experiments imply that intestinal permeability is regulated by DP1 expressed on the apical side of epithelial cells.

Intestinal permeability is an important factor contributing to intestinal inflammation and food allergies. Several studies have reported that patients with IBD exhibit increased intestinal permeability (19, 20). Raju et al. showed that the administration of a claudin-2 inactivator improved immune-mediated colitis, followed by decreased intestinal permeability in mice (5). Moreover, Ventura et al. showed that intestinal permeability increased in individuals with food allergies and was positively correlated with symptom severity (21). These reports demonstrated that intestinal permeability is a promising therapeutic target for intestinal disorders. In the present study, we revealed that pharmacological DP1 stimulation decreased intestinal permeability both *in vivo* and *ex vivo*. This observation reinforces the previously reported therapeutic effects of DP1 agonists on colitis/food allergies (8, 9).

Mucus acts as a physiological barrier between epithelial cells and intestinal lumen. Using a horizontal imaging chamber, Ermund et al. showed that beads the size of bacteria did not penetrate the mucus layer of resected mouse colons (22). Other studies have shown that treatment with a mucolytic agent, NAC, increases the intestinal permeability of dextran, both *ex vivo* and *in vivo* in rats (23, 24). In the current study, pharmacological DP1 stimulation induced mucus secretion, and the removal of this mucus canceled the suppressive effect of the DP1 agonist on intestinal permeability. Therefore, DP1-induced mucus secretion is likely to decrease intestinal permeability by limiting the physiological contact of FD40 with epithelial cells.

Goblet cells store synthesized mucin proteins within the granules and, upon stimulation, release the granules through exocytosis. DP1 couples with Gs, which stimulates adenylyl cyclase and increases intracellular cAMP levels. Kuver et al. showed that cAMP-increasing reagents including PGE_2_ and epinephrine, stimulate mucus secretion in dissected mouse intestines (18). Based on these observations, it is reasonable to conclude that the DP1 agonist stimulates mucus secretion by increasing intracellular cAMP levels. Although the mechanism underlying cAMP-induced mucus secretion remains elusive, several studies have revealed the cAMP-induced exocytotic process in other secretory cells, including neurons and pancreatic β-cells. This process is mediated by both protein kinase A and Epac-dependent mechanisms (25), the two known downstream signals of cAMP. Further studies are needed to clarify the downstream signaling of cAMP that triggers mucus secretion following DP1 stimulation.

There are several other potential targets of DP1 agonists that enhance the intestinal barrier. The present study showed that DP1 agonism did not alter the TER of Caco-2 monolayer for 30 min at least under the experimental conditions. Consistently, Rodríguez-Lagunas et al. reported that the administration of EP2 and EP4 agonists, two cAMP-increasing reagents, for 3 h did not alter the TER of Caco-2 cells (26). In contrast, another study showed that the administration of another cAMP-increasing IP agonist for 24 h increased the TER of Caco-2 cells (4). Based on these findings, the DP1 agonist potentially enhances tight junction integrity for a longer time. Intestinal wound repair is another important mechanism for barrier maintenance, especially during impairment. cAMP-increasing agents including PGE_2_ promote intestinal repair (27), suggesting that DP1 agonists also promote this reaction. A previous study also revealed that a DP1 agonist enhanced vascular endothelial barrier function *in vivo* in mouse ears (28). This finding gives rise to the possibility that the DP1 agonist also enhanced intestinal vascular endothelial barrier, and consequently limited FD40 migration to the bloodstream. Thus, DP1 agonists potentially exert various protective effects on intestinal barrier function. Further studies are required to elucidate the mechanism of action of this agonist.

This study focused on the effects of an exogenous DP1 simulation. PGD_2_ is produced in the normal mouse intestine (27), and its level increases in a food-allergic mouse model (29). Moreover, the administration of BWA868C, a DP1 antagonist, exacerbated food allergy symptoms in a mouse model (9). These studies show that endogenously produced PGD_2_ activates DP1 in the pathological state and potentially protects the barrier by promoting mucus secretion. In the present study, an apically administered DP1 agonist directly stimulated DP1 expression on the apical side of the epithelial cells in the *ex vivo* permeability assay. In contrast, intraperitoneally administered DP1 agonists *in vivo* probably circulated in the bloodstream and stimulated intestinal epithelial cells. We could not determine how the circulating DP1 agonist stimulated intestinal epithelial cells. Since DP1 is expressed on the apical side of epithelial cells, this receptor may function as a sentinel to monitor luminal contents. Further studies are needed to elucidate the biological significance of DP1 signaling regarding physiological ligands and their sources and distribution.

In conclusion, we revealed that pharmacological DP1 stimulation decreases the intestinal permeability of dextran conceivably through mucus secretion. This is potentially an additional mechanism underlying the therapeutic effects of DP1 agonists on colitis and food allergies.

## Data availability statement

The original contributions presented in the study are included in the article/supplementary material. Further inquiries can be directed to the corresponding author.

## Ethics statement

The animal study was approved by The Institutional Animal Care and Use Committee of the University of Tokyo. The study was conducted in accordance with the local legislation and institutional requirements.

## Author contributions

AH: Investigation, Writing – original draft, Data curation, Formal Analysis, Methodology. NS: Data curation, Formal Analysis, Investigation, Methodology, Writing – review & editing. KK: Data curation, Formal Analysis, Writing – review & editing, Writing – original draft. TM: Writing – original draft, Writing – review & editing, Conceptualization, Funding acquisition, Investigation, Project administration, Resources, Supervision.
